# Comprehensive Insights of Biochemical and Sensory Characterization in *Takifugu obscurus* by Environmental Modulation

**DOI:** 10.3390/foods14081386

**Published:** 2025-04-17

**Authors:** Siman Li, Xuanyun Huang, Changling Fang, Yongfu Shi, Xiaoyi Lou, Dongmei Huang, Yunyu Tang

**Affiliations:** Ministry of Agriculture and Rural Affairs, East China Sea Fisheries Research Institute, Chinese Academy of Fishery Sciences, Jungong 300, Shanghai 200090, China; lism@ecsf.ac.cn (S.L.); hxyseven@163.com (X.H.); fangcl@ecsf.ac.cn (C.F.); xyzmn530@sina.com (Y.S.); huoxingmayi@126.com (X.L.); hdm2001@126.com (D.H.)

**Keywords:** *Takifugu obscurus*, pufferfish, salinity, season, fasting, sensory, nutrition

## Abstract

This study systematically deciphers a comprehensive analysis of biochemical and sensory variations in *Takifugu obscurus*, evaluating the seasonal dynamics, salinity gradients (0 and 3‰), and nutritional regimes on quality determinants in aquaculture. To the best of our knowledge, the mechanistic links between cultivation factors and organoleptic quality were first established through integrative biochemical profiling, including proximate composition, free amino acids, taste-active nucleotides, and mineral ions, coupled with quantitative sensory evaluation. The results revealed that spring samples exhibit 44.7% higher inosine monophosphate than that of autumn and 92.8% elevated ATP, correlating with superior umami-kokumi attributes. Salinity adaptation drove metabolic trade-offs: freshwater cultivation amplified flavor-enhancing amino acids, while brackish systems prioritized ionic precision. Short-term fasting induced alanine accumulation without sensory compromise, demonstrating nutritional plasticity. Polyculture compatibility was evidenced by negligible quality divergence from monoculture, despite enhanced productivity. These insights advance sustainable aquaculture through science-driven strategies that harmonize ecological resilience, economic viability, and culinary excellence in commercial *Takifugu obscurus* production.

## 1. Introduction

*Takifugu obscurus*, commonly known as the dark-spotted pufferfish, is a significant species in East Asian cuisine and aquaculture. Globally, China contributes approximately 70% of the global pufferfish supply, most of which is exported internationally [1,2]. The legalization of its consumption in China has greatly heightened its commercial and culinary values due to its unique taste and nutritional benefits [3,4,5].

Aquaculture practices play a crucial role in determining fish quality, with environmental factors such as water salinity, seasonal changes, and feeding practices significantly influencing the biochemical composition and sensory characterization of fish [6]. Despite its economic importance, existing studies on *T. obscurus* predominantly focus on isolated environmental factors and their effects on growth performance or biochemical composition [7,8,9]. However, a critical knowledge gap remains in understanding how the synergistic interactions of multiple environmental variables collectively shape both biochemical traits and sensory attributes of *T. obscurus*. This gap hinders the development of holistic aquaculture strategies to optimize flavor quality—a key determinant of consumer preference and market value [10,11]. Given the species’ expanding role in global seafood trade, addressing this gap is imperative to align aquaculture practices with industry demands for high-quality, sustainably farmed pufferfish.

The taste and overall sensory quality of fish are closely tied to its biochemical composition [12]. Free amino acids, taste-active nucleotides, and mineral ions are key components that define the flavor profile of aquatic species [2,4,5,7]. Nonetheless, the mechanisms underlying the effects of aquaculture conditions on these components in *T. obscurus* remain insufficiently studied. Japanese researchers documented the effects of salinity water on cultivated pufferfish, finding that samples from salinity water groups received higher scores in sensory evaluations [8]. Gwang-Yeol Yoo et al. [9] analyzed the relationship between feeding frequency, water temperature, stocking density, and pufferfish growth, but their study only examined environmental factors related to fish body weight without discussing nutritional composition or sensory attributes. Liu et al. [13] investigated the differences in taste-active compounds between *Takifugu obscurus* and *Takifugu rubripes*, demonstrating that inosinic acid (IMP) is a major flavor compound, and Na and K ions make significant contributions to pufferfish taste. However, they did not explore whether these differences were determined by aquaculture environments.

While previous studies have examined either biochemical components or environmental factors, respectively, multidimensional analytical research is necessary to thoroughly assess *T. obscurus* quality [14,15,16]. To systematically address the underexplored interactions between multifactorial environmental variables and T. obscurus quality traits, this study integrates comprehensive biochemical profiling with sensory evaluation across diverse aquaculture environments. By analyzing samples collected from representative farms in Guangdong Province, we investigate how seasonal dynamics, salinity gradients (0 and 3‰), and nutritional regimes collectively shape flavor-related compounds and further correlate these biochemical signatures with sensory attributes such as umami-kokumi synergy. Through this multidimensional approach, the research aims to establish a scientific foundation for optimizing T. obscurus quality, with direct implications for sustainable aquaculture practices, nutritional enhancement strategies, and gastronomic innovation. The findings are designed to provide actionable strategies for optimizing harvest timing, salinity adaptation, and feeding practices, thereby bridging the gap between aquaculture sustainability and market-driven quality standards.

## 2. Materials and Method

### 2.1. Chemicals

The standards of free amino acids, including aspartic acid (Asp), glutamic acid (Glu), serine (Ser), glycine (Gly), threonine (Thr), alanine (Ala), proline (Pro), arginine (Arg), lysine (Lys), valine (Val), histidine (His), tyrosine (Tyr), phenylalanine (Phe), isoleucine (Ile), leucine (Leu), cysteine (Cys), methionine (Met), nucleotides, including cytidine monophosphate (CMP), guanosine monophosphate (GMP), inosinic acid (IMP), adenosine triphosphate (ATP), adenosine diphosphate (ADP), adenosine monophosphate (AMP), hypoxanthine (HX), inosine (HXR1), NaCl, monosodium glutamate, sucrose, and citric acid, were all supplied by Dr. Ehrenstorfer (Augsburg, Germany). The standard solutions of sodium and potassium were purchased from Agilent Technologies Co., Ltd. (Santa Clara, CA, USA). Chromatographic grade acetonitrile, n-hexane, methanol, and formic acid were supplied by J. T. Baker Chemical Co. (Phillipsburg, NJ, USA). Ammonium acetate, NaOH, nitric acid, perchloric acid, and ammonium carbonate were analytical grade and supplied by Sinopharm Group Chemicals Limited (Shanghai, China). The C18 solid phase extraction cartridges were purchased from Agela Technologies (Tianjin, China).

### 2.2. Sample Collections

All experimental *T. obscurus* pufferfish were collected from aquaculture facilities in Guangdong Province, China. The sampling plan included two seasonal periods. The juvenile pufferfish weighing 150–200 g were raised for 6 months from April to October for the autumn group and from November to May for the spring group. For each group, pufferfish were cultivated under three different water conditions: freshwater (0‰ salinity), saline water (3‰ salinity), and a mixed farming system with grass carp (3‰ salinity). Prior to sampling, each environmental group was further divided based on pre-harvest feeding management. One subgroup was maintained on a regular feeding schedule, while the other was subjected to a 5-day fasting period. This resulted in a total of twelve distinct experimental groups (Table 1).

The caught *T. obscurus* was slaughtered according to the National Standard (GB/T 27624–2011 [17]) by professional pufferfish operators. After that, the dorsal muscle of fish flesh was cut off, packaged, and transported to our lab within 12 h covered with ice, and frozen at −80 °C until use.

### 2.3. Preparation of T. obscurus

Frozen fillets were thawed at 4 °C overnight. All samples were tested for the presence of tetrodotoxin [3] and found to meet the criteria for edibility. The fillets were divided into two groups: those intended for sensory evaluation were cut to a similar weight (10 ± 1.00 g, 10.0 ± 0.5 cm length, 2.5 ± 0.5 cm width) from the same part of the samples before cooking. After that, they were steam-cooked uniformly in water for 10 min. Raw fillets designated for biochemical analysis were homogenized and stored at −80 °C until further processing.

### 2.4. Quantitation of Proximate Composition

#### 2.4.1. Crude Protein

The protein content was determined using the Dumas method with a nitrogen analyzer LECO [18]. To ensure the precision of the nitrogen content measurement, three replicates of approximately 1 g of each sample were analyzed. The crude protein content of all samples was subsequently calculated using a nitrogen-to-protein conversion factor of 6.25, as reported by Mariotti [19].

#### 2.4.2. Crude Lpid

Crude lipids were extracted from the samples using a mixture of chloroform and methanol (2:1, *v*:*v*), as described in previous research [20]. Portions of 3 mL from the bottom phase were measured into pre-weighed dry glass tubes, and the chloroform was evaporated under mild conditions at ambient temperature using N_2_. The dried samples were then weighed to calculate the crude lipid content.

#### 2.4.3. Ash and Moisture

Ash content was determined following the procedure by Sluiter [21]. This method is typically expressed as the percentage of residue remaining after drying at a temperature range of 550 °C to 600 °C.

Moisture content was assessed according to the previous report [22]. Initially, aluminum dishes were pre-dried at 105 ± 3 °C for approximately 4 h. The dishes were then cooled in a desiccator and weighed to the nearest 0.1 mg. Samples were placed in the pre-dried dishes and then dried in the oven at 105 ± 3 °C for 24 h to determine the mass of water lost during the drying process.

### 2.5. Quantitation of Free Amino Acid

Liquid chromatography-mass spectrometry was performed using a Waters TQ-XS system with electrospray ionization. Amino acids were extracted via formic acid/water/methanol system extraction without a derivatization step [23]. Separation was achieved on a C18 column (2.1 × 100 mm, 1.7 μm) with a gradient elution profile. Multiple reaction monitoring mode was employed for quantitative analysis, with external standards for precision validation.

### 2.6. Quantitation of Nucleotides

High-performance liquid chromatography (HPLC) analysis of nucleotides was conducted using an Agilent 1200 system equipped with a UV–vis detector. Nucleotides were extracted via perchloric acid precipitation and neutralization. Separation was performed on a reversed-phase C18 column with a mobile phase gradient of acetonitrile and phosphate buffer [24]. Quantification was based on external standard calibration curves.

### 2.7. Quantitation of Inorganic Ions

Quantitative determination of sodium (Na^+^) and potassium (K^+^) ions was conducted via flame atomic absorption spectroscopy (ZEEnit 700, Analytik Jena AG, Jena, Germany) in strict adherence to AOAC International Official Method [25]. The analytical protocol included wavelength calibration at 589.0 nm (Na^+^) and 766.5 nm (K^+^), with matrix-matched calibration standards spanning 0.1–10.0 ppm.

### 2.8. Sensory Evaluation

A total of 30 sensory evaluation participants were recruited, maintaining gender balance (15 males and 15 females) with an age distribution ranging from 21 to 60 years. Prior to the formal sensory evaluation experiment, all 30 assessors underwent rigorous proficiency testing according to standardized protocols, with comprehensive qualification verification [ISO 8589:2007(E)] [26]. Subsequently, intensive specialized training was conducted, focusing on aquatic product flavor characteristics and enhancing participants’ capabilities in fresh fish flavor identification and evaluation [27].

The study employed a five-dimensional sensory evaluation approach: umami, sweetness, saltiness, kokumi, and fishy flavor. Scoring system: The 0–8 point scoring system was implemented; 7–8 points: extremely strong; 5–6 points: strong; 3–4 points: average; 1–2 points: weak; 0 points: very weak or none. The intensity of ultrapure waters was set as point 0. And 5 points defined by reference chemical solutions (0.595 mg/mL monosodium glutamate for umami, 0.430 mg/mL citric acid, 1.19 mg/mL NaCl, 5 mM glutathione, and 10 mM trimethylamine for fishy flavor). Panelists underwent blind training to calibrate their perception against these references. This approach standardizes subjective perception through objective chemical anchors. Each sample set was independently evaluated by assessors using standardized evaluation sheets, recording scores for each dimensional parameter.

### 2.9. Statistical Analysis

Statistical analyses were conducted using IBM SPSS Statistics (Version 20.0, SPSS, Inc., Chicago, IL, USA). Prior to multivariate analysis, dataset normalization was conducted through Z-score standardization in SPSS to ensure comparability of variables. Coefficient of variation (CV = SD/Mean × 100%) was adopted to standardize data dispersion, as the variables exhibited significant differences in units and scales. Group differences were evaluated using one-way analysis of variance (ANOVA) followed by Tukey’s post hoc test (α = 0.05). Principal component analysis (PCA) was implemented in SPSS with varimax rotation to identify multivariate patterns in the normalized data. Correlation structures were examined using Pearson’s correlation coefficients calculated in SPSS. Visualization of analytical results was performed using specialized graphical tools: Column charts and PCA biplots were created using GraphPad Prism (Version 10.2.0; GraphPad Software, Boston, Massachusetts, USA), while correlation heatmaps and radar charts were generated through ChiPlot (https://www.chiplot.online/).

## 3. Results and Discussion

### 3.1. Distribution and Content of Parameters

The analysis of the twelve experimental groups can be seen in Table 2, which revealed substantial variations in the concentrations of moisture, ash, crude protein, crude lipid, free amino acids, nucleotides, and inorganic ions in *T. obscurus*. The range and coefficient of variation (CV) for each parameter were calculated to assess the variability across different aquaculture conditions [28].

As shown in Table 2, moisture content was relatively stable across the groups, ranging from 74.17% to 79.25% with a CV of 2.20%. Ash content exhibited moderate stability across samples, with a range of 3.38% to 4.39% and a CV of 8.45%, indicating limited sensitivity to aquaculture factors. Crude protein levels were observed in the range of 17.60–22.46% (8.17% of CV), reflecting a moderate degree of variability in this parameter. In contrast, significant variability was observed in crude lipid content, ranging from 0.60% to 2.10% with a CV of 39.63%, suggesting significant sensitivity to environmental and nutritional factors. A similar result was reported by Fallah et al. [29], who observed that muscle moisture and total lipid contents in *Capoeta damascina* were significantly affected by salinity, suggesting considerable sensitivity to environmental factors. Also, this finding aligns with Romano et al.’s research [30], demonstrating that salinity influences protein content in *Macrobrachium rosenbergii*. Furthermore, Sergio et al. [31] confirmed that both temperature and salinity significantly impact protein and lipid contents in *Lutjanus guttatus*, reinforcing the notion that aquatic species exhibit marked sensitivity to environmental factors. The results showed that the environmental factors primarily influence the proximate composition of *T. obscurus*, specifically the crude lipid content. As *T. obscurus* is recognized as a low-fat fishery resource, its lipid content constitutes an essential quality evaluation. The significant dispersion in crude lipid values (CV = 39.63%) provides empirical evidence for environmental mediation of tissue composition in this species.

Among the free amino acids, Pro showed the highest CV of 88.85%, indicating a great sensitivity to changes in aquaculture conditions. Gly and Lys exhibited moderate variability with CV of 26.64% and 23.54%, respectively, aligning with the established patterns of fish amino acids under variable environmental and nutritional regimes, displaying considerable variation with CVs of 26.64% and 23.54%, respectively. These variations are consistent with the typical biochemical responses of fish to different environmental and feeding conditions [32,33,34,35]. Glu concentration was observed in the range of 0.348–2.187 mg/100 g, with a CV of 60.69%, showing significant influence on gustatory perception associated with umami characteristics [5]. This was corresponding to the current result that free amino acid content in sardines fluctuates markedly with seasonal changes by Šimat et al. [36]. Similarly, Wei and Liu [37] reported that varying cultivation salinity levels significantly affect both free amino acid and nucleotide contents in *Pacific oysters*. In the nucleotide fraction, IMP content in the samples ranged from 220.480 to 398.780 mg/100 g (CV of 19.70%), exhibiting a notable variation as a key mediator of umami taste potentiation. This nucleotide is crucial for umami taste development [38]. AMP had the highest CV among nucleotides at 52.17%, indicating dynamic changes in energy metabolism [39]. Additionally, ions are essential for osmoregulation and can influence the overall taste profile. Inorganic ion analysis demonstrated moderate variability, with Na^+^ concentrations quantified in the range of 34.550–56.290 mg/100 g (CV of 15.83%) and K^+^ at 170.280–307.800 mg/100 g (CV of 17.65%). These ions play critical roles in osmoregulatory homeostasis and sensory quality modulation through ionic strength effects on macromolecular conformation [40].

These results indicated the substantial modulation of *T. obscurus* in biochemical constitution and sensory properties by environmental and nutritional variables. Specifically, metabolic biomarkers, including crude lipids, Pro, AMP, and Glu, exhibited pronounced variability (CVs: 39.63–88.85%), indicative of their acute responsiveness to aquaculture factors. While moisture, ash, and crude protein demonstrated superior stability (CVs: 2.20–8.45%), reflecting relatively stable conditions under varying environmental regimes. These findings highlight the complex interplay between environmental factors and fish quality, suggesting that targeted regulation of key aquaculture parameters is effective in enhancing both gustatory perception and nutrient retention efficiency in *T. obscurus*. Management of these variables is essential for optimizing the sensory and nutritional qualities of pufferfish.

### 3.2. Correlation Analysis Between Chemical Components and Sensory Attributes

**Figure 1:**
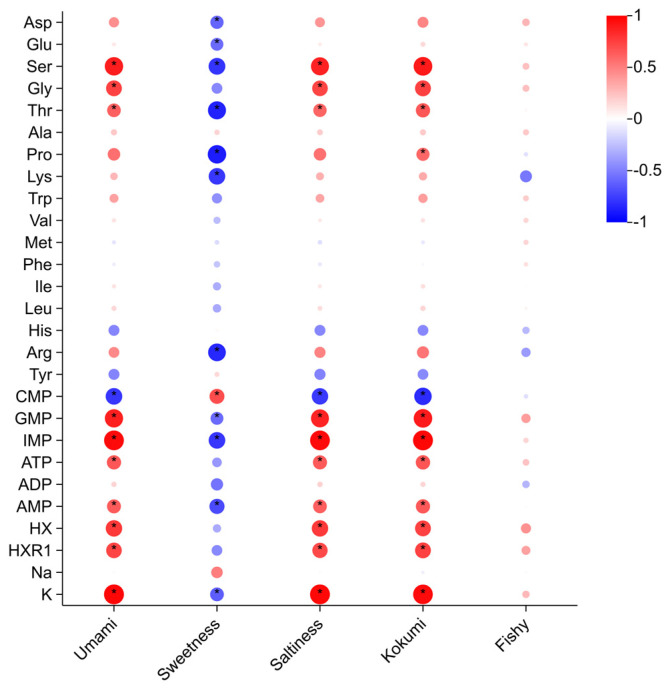
Correlation maps of chemical components and sensory attributes. (* *p* < 0.05 vs. raw).

It is essential to elucidate the multifaceted interactions between biochemical constituents and sensory modalities in *T. obscurus*, with a specific focus on quantitative associations with umami, sweetness, saltiness, kokumi, and piscine flavor attributes. Through the application of correlation analysis (Figure 1), we employed *p*-value thresholds and Pearson’s correlation coefficients (r) to assess both statistical significance and interaction magnitude.

The taste of umami in *T. obscurus*, a defining sensory trait to its gastronomic value [6], was predominantly potentiated by synergistic interactions between specific amino acids and nucleotides. Ser and Gly exhibited an obviously significant positive correlation with umami intensity (r = 0.89, *p* < 0.001 and r = 0.74, *p* < 0.01, respectively), suggesting its role as a primary umami-enhancing modulator. Notably, IMP emerged as the dominant umami mediator (r = 0.96, *p* < 0.001), consistent with its established role in nucleotide-amino acid synergy within umami taste transduction cascades. These findings highlight the importance of these specific compounds in contributing to the umami taste profile of the pufferfish.

Sweetness, another important sensory attribute, was significantly affected by several amino acids. As shown in Figure 1, Ser and Pro both showed highly significant negative effects on sweetness (*p* < 0.01, r = −0.80 and *p* < 0.01, r = −0.89, respectively), suggesting that the higher concentrations of these amino acids might reduce the perception of sweetness in *T. obscurus*. Interestingly, Ala had a non-significant positive correlation with sweetness (*p* > 0.05, r = 0.17), while Lys exhibited a significant negative correlation (*p* < 0.01, r = −0.78). These differential modulations reveal that a complex interplay between these compounds and sweetness in *T. obscurus* is due to the bidirectional amino acid signaling in sweet taste chemoreception.

Saltiness perception in *T. obscurus* was predominantly regulated by synergistic interactions between amino acids and inorganic ions (Figure 1). Ser and Gly both showed significant positive correlations with saltiness intensity (*p* < 0.01, r = 0.87 and *p* < 0.01, r = 0.73, respectively), suggesting their potential roles in enhancing it attribute. K^+^ also presents a highly significant positive correlation with saltiness (*p* < 0.001, r = 0.97), consistent with its established function in potentiating salt taste. Although not statistically significant, Pro and His showed notable correlations with saltiness (*p* > 0.05, r = 0.56 and *p* > 0.05, r = −0.48, respectively), suggesting potential auxiliary roles in salt taste modulation.

Kokumi, a taste attribute associated with richness and mouth fullness [41,42,43]. It was significantly influenced by several biochemical components. Ser and Gly both exhibited highly significant positive correlations with kokumi taste (*p* < 0.001, r = 0.90 and *p* < 0.01, r = 0.75, respectively). IMP also showed an obviously strong positive correlation with kokumi (*p* < 0.001, r = 0.97). Additionally, CMP and GMP demonstrated significant correlations with kokumi (*p* < 0.01, r = −0.83 and *p* < 0.001, r = 0.89, respectively). These results indicated that the nucleotides contribute to the overall kokumi perception.

The fishy taste, a sensory attribute frequently associated with consumer aversion in aquatic products [44], demonstrated limited significant correlations with the majority of biochemical constituents. However, certain compounds like HX and HXR1 exhibited the relatively weaker positive correlations with fishy taste (*p* < 0.01, r = 0.43 and *p* < 0.01, r = 0.37, respectively). This suggests that while these components may influence the fishy taste, their impact is relatively minor compared to other sensory attributes.

The correlation analysis elucidated the intricate metabolic–sensory interactions impact on *T. obscurus* quality, offering valuable insights into the biochemical determinants of sensory properties. These findings establish a scientific foundation for precision aquaculture strategies aiming at optimizing nutrient formulation to enhance desirable taste attributes such as umami and sweetness while minimizing off-flavors like fishy taste through environmental parameter adjustments. The robust associations between specific amino acids, nucleotides, and inorganic ions with taste modalities underscore the feasibility of targeted interventions. These investigation insights not only advance our understanding of flavor biochemistry in *T. obscurus* but also provide actionable strategies for improving its commercial value.

### 3.3. Environmental Factor Stratification

#### 3.3.1. Insights into Seasonal Variations

**Figure 2:**
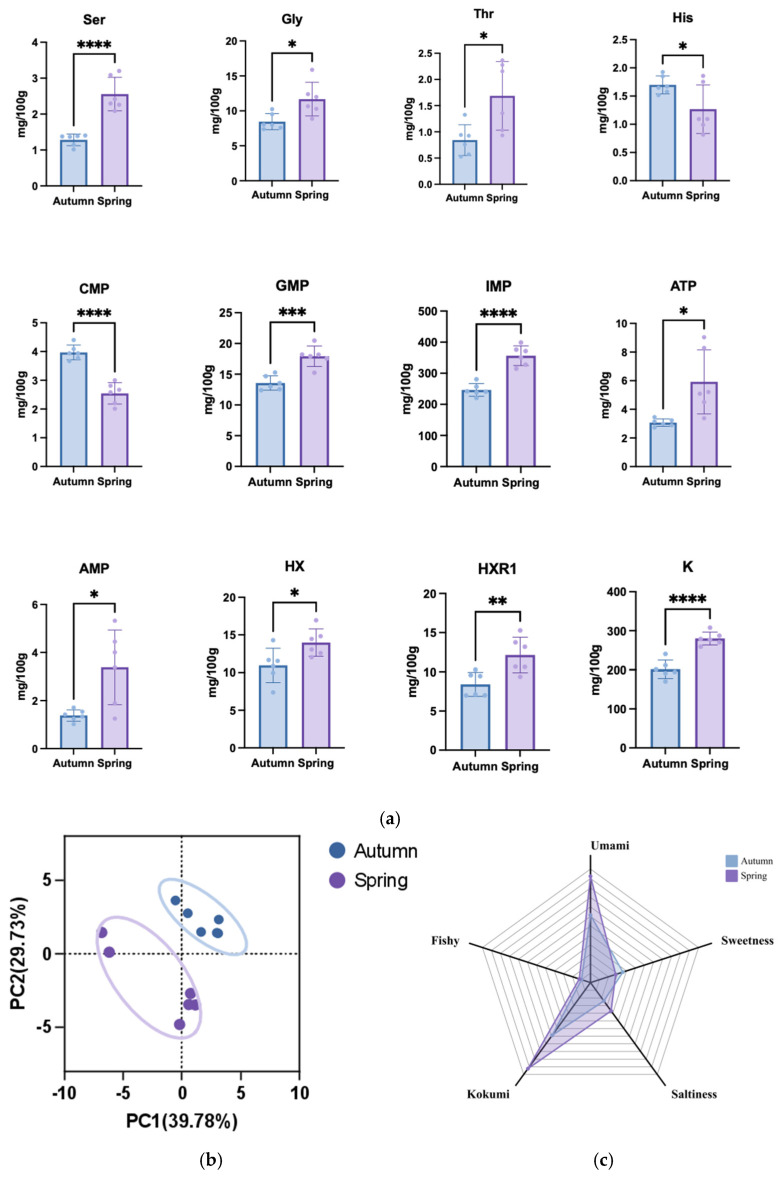
Analysis in *T. obscurus* for different seasons. (**a**) The contents of free amino acid, nucleotide, and inorganic ion (* *p* < 0.05, ** *p* < 0.01, *** *p* < 0.005, **** *p* < 0.0001, vs. raw); (**b**) PCA; (**c**) sensory evaluation.

Seasonal variations induced marked metabolic divergence in *T. obscurus*, with spring and autumn samples exhibiting distinct biochemical profiles originating from the overwintering aquaculture strategy. A cold-induced adaptive process enhancing nutritional quality and flavor complexity through metabolic remodeling was observed in *T. obscurus*, which undergoes an overwintering period during the growth process [45]. While Eriocheir sinensis [46] shows post-overwintering muscle texture enhancement coinciding with selective amino acid depletion. This contrasts with Scylla paramamosain [47], where overwintering triggered 20% crude protein loss but elevated umami characteristics, revealing a fundamental trade-off between nutritional preservation. The contents of differential free amino acids, nucleotides, and inorganic ions detected in samples from different seasons were shown in Figure 2. As delineated in Figure 2a, the spring samples exhibited significant upregulation of gustatory amino acids, notably Ser (2.56 vs. 1.28 mg/100 g) and Gly (11.68 vs. 8.46 mg/100 g), which serve dual functions by potentiating sweet taste perception and providing cryoprotective osmoregulation during cold adaptation. The metabolic tendency suggests the overwintering period induces a survival–flavor trade-off, where amino acid accumulation simultaneously buffers cellular freeze stress and sensory appeal, offering insights for seasonally optimized aquaculture practices. Nucleotide profiles emerged as sensitive biomarkers of variation, with IMP levels 1.45-fold higher in spring *T. obscurus* than in autumn (356.52 vs. 246.36 mg/100 g, *p* < 0.0001), consistent with cold-acclimatized nucleotide accumulation patterns observed across other aquaculture species [48]. The spring samples exhibited umami potential arising not only from IMP-GMP synergy (GMP: +32.2%) but also through ATP-driven freshness preservation (ATP: +92.8%). The higher ATP levels (5.92 vs. 3.07 mg/100 g) in spring samples suggest the metabolic prioritization of freshness-related nucleotides during overwintering to adapt to the seasonal variations. Additionally, K+ modulation (280.00 vs. 201.39 mg/100 g, *p* < 0.01) reflects sophisticated adaptive osmoregulation established as a strategy convergent with euryhaline osmolytes allocation principles. This integrated metabolic-osmotic adjustment for seasonal quality optimization was observed in aquatic species [49].

PCA provided multivariate validation of quantified season-specific metabolic states, demonstrating significant segregation of spring and autumn groups across PC1 (52.3%) and PC2 (17.4%), which collectively explained 69.71% of total variance (Figure 2b). The separation was principally driven by Asp (0.89), Ser (0.85), and Trp (0.78), whose coordinated accumulation in spring specimens suggests synchronized regulation of amino acid metabolism during overwintering. The tighter clustering of spring samples (intra-group variance reduction of 43% vs. autumn) indicates more consistent biochemical profiles through post-overwintering, effectively buffering environmental stochasticity. The biochemical divergence translated into seasonally distinct sensory phenotypes (Figure 2c), with spring *T. obscurus* exhibiting enhanced gustatory properties of umami (scores of 5.62 vs. 3.57) and kokumi (scores of 5.62 vs. 3.45) intensities. This sensory superiority can be attributed to several synergistic metabolic adaptations. Flavor-enhancing synergy between upregulated free amino acids (FAA) and 5′-nucleotides creates the optimal umami potentiation development. Energy state preservation via maintained ATP/ADP ratios suggests the structural integrity of muscle tissue, and the coordinated FAA-K^+^ accumulation contributes to optimal cellular hydration. These findings align with prior reports on taste-active compound dynamics in cold-adapted fish [50,51].

The seasonal variations were observed in spring *T. obscurus* with sophisticated metabolic adaptations and quality optimization. The biochemical profile of spring samples suggests activation of multiple adaptive pathways, including enhanced energy metabolism, sophisticated osmoregulatory responses, and optimized flavor precursor development during cold adaptation. These results provide actionable insights for aquaculture innovation, including synchronizing harvests with metabolic peaks (post-overwintering) and implementing nucleotide/amino acid profiling for quality control.

#### 3.3.2. Insights into Salinity Conditions

**Figure 3:**
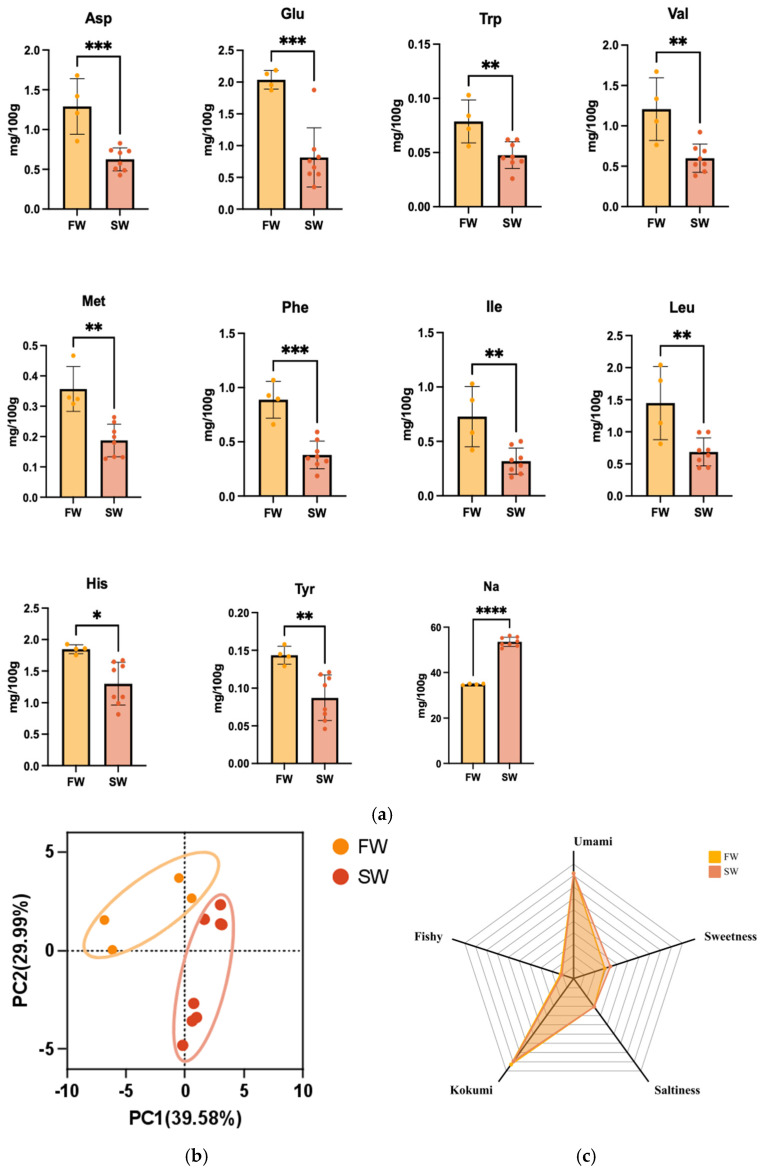
Analysis in *T. obscurus* for different salinity. (**a**) The contents of free amino acid, nucleotide, and inorganic ion (* *p* < 0.05, ** *p* < 0.01, *** *p* < 0.005, **** *p* < 0.0001, vs. raw); (**b**) PCA; (**c**) sensory evaluation.

Low-salinity culture systems dominate pufferfish aquaculture [52], while marine-cultured congeners (*F. flavidus*, *F. rubripes*) demonstrate superior flavor metrics versus *T. obscurus* [1], implicating salinity-specific metabolic adaptations in taste compound biosynthesis. The salinity contrast (0‰ vs. 3‰) on *T. obscurus* cultivation revealed complex osmoregulatory and metabolic adaptation mechanisms. Analysis of the relative biochemical cartography demonstrated distinct patterns between this cultivation condition, as illustrated in Figure 3.

The significant amino acid accumulation was observed in *T. obscurus* under salinity variations (Figure 3a), with freshwater cultivation driving Asp (1.29 vs. 0.62 mg/100 g), Glu (2.04 vs. 0.82 mg/100 g), and branched-chain amino acids (Val, Ile, and Leu collectively 2.98 vs. 1.42 mg/100 g) increased by 108–148% compared to saline counterparts. This hyperaccumulation aligns with findings in other euryhaline aquatic species where environmental salinity modulates amino acid metabolism as part of osmotic adaptation, a process driven by the dual role of amino acids as both osmolytes and flavor mediators. In low-salinity conditions, organisms such as swimming crabs exhibit selective retention of umami-associated amino acids while reducing total free amino acid pools [53], suggesting a metabolic prioritization strategy that balances osmotic demands with sensory quality. This phenomenon extends across aquatic taxa, as demonstrated by systemic upregulation of essential amino acid biosynthesis pathways under salinity fluctuations [49], where amino acids not only counteract ionic gradients but also enhance flavor profiles through compositional shifts. The adaptive synergy is further exemplified in saline-alkaline environments, where organisms maintain osmotic equilibrium through elevated free amino acid content while simultaneously improving umami perception via coordinated interactions between retained amino acids and flavor nucleotides [54]. Such metabolic rewiring underscores a conserved biological logic—environmental salinity acts as a biochemical modulator, orchestrating amino acid dynamics that concurrently stabilize cellular homeostasis and optimize gustatory characteristics through selective accumulation and catabolic regulation. The elevated levels of these amino acids in freshwater samples suggest enhanced protein metabolism and potential compensatory mechanisms for maintaining cellular osmolarity. In contrast, nucleotide compositions remained conserved across salinity regimes, with IMP marginal variation (318.10 vs. 293.10 mg/100 g). While GMP showed slightly higher levels in freshwater samples (16.58 vs. 15.34 mg/100 g). These results suggest salinity-independent maintenance of energy metabolism for stabilizing postmortem freshness. The most obvious environmental signature emerged in mineral ion profiles, where freshwater samples exhibited 53% reduced Na^+^ content (34.88 vs. 53.53 mg/100 g, *p* < 0.0001), directly reflecting external salinity influence on osmoregulatory mechanisms [55]. The differential ion accumulation suggests sophisticated osmoregulatory adaptations that may influence both physiological function and taste characteristics. Such compartmentalized adaptation not only preserves cellular homeostasis but also modulates taste perception, which may be ascribed to the fact that the elevated Na^+^ in saline environments enhances saltiness, whereas freshwater amino acid surges potentiate umami-kokumi synergy.

The multivariate biochemical PCA analysis exhibited distinct environmental signatures (Figure 3b), revealing clear segregation between cultivation environments, along with the first two principal components explaining 69.57% of the total variance. The loading plot identified sodium content and amino acid concentrations as the primary discriminating determinants (loading values of 0.72 and −0.95 for PC1, respectively). The inverse covariance (−0.83, *p* < 0.001) between these two factors reflects fundamental trade-offs in osmoregulatory strategy: ionic dominance in saline versus organic osmolyte reliance in freshwater systems. The broader dispersion of saline samples in the PCA plot suggests enhanced metabolic variability under osmotic stress conditions. Complementary sensory evaluation exhibits the environment-specific taste profiles to perceptible quality parameters (Figure 3c). The saline-adapted samples demonstrated 28% elevated saltiness scores (1.53), but 3.7% reduced kokumi intensity (4.48 vs. 4.65). It is worth noting that umami perception remained relatively consistent across environments (4.59 vs. 4.60), suggesting that fundamental taste characteristics, potentially nucleotide stabilization (IMP: 318.10 vs. 293.10 mg/100 g) and glutamate–glutamine cycling buffer core flavor attributes against osmotic perturbations. The observed flavor dynamics in saline-adapted samples reveal a nuanced regulatory mechanism where saltiness enhancement and kokumi modulation occur independently of core umami stability—a phenomenon reflecting specialized evolutionary adaptations to osmotic environments. While elevated saltiness perception aligns with known ion-channel sensitization pathways, such as the potential activation of calcium-sensing receptors and transmembrane channel-like 4 by charged peptides under salinity stress [40], the selective preservation of umami characteristics likely stems from dual biochemical safeguards: nucleotide stabilization maintaining IMP thresholds critical for taste receptor activation [56], coupled with glutamatergic cycling that buffers against amino acid pool fluctuations through coordinated glutamate–glutamine metabolism [57]. Notably, the divergence between saltiness amplification and kokumi attenuation suggests a metabolic prioritization strategy observed across euryhaline species, where organisms optimize ionic signaling efficiency at the expense of secondary taste complexity—a pattern exemplified in largemouth bass muscle texture refinement and IMP retention under salinity gradients. This adaptive trade-off extends current understanding by demonstrating taste modality-specific resilience—a regulatory hierarchy where evolutionarily conserved umami perception resists environmental perturbation through nucleotide-amino acid synergy, while ancillary flavor dimensions become tunable variables modulated by salinity-driven metabolic rewiring [40,56,57].

The relationship between environmental salinity and biochemical composition reveals sophisticated adaptive mechanisms that extend beyond simple osmotic responses. These findings establish salinity as a master regulator of both physiological resilience and culinary quality, informing targeted aquaculture strategies to optimize environmental parameters for dual metabolic–taste outcomes. Meanwhile, environmental salinity as a tunable parameter for precision flavor engineering in aquaculture enables the targeted modulation of taste receptor activation landscapes through controlled metabolic steering.

#### 3.3.3. Insights into Nutritional Regime

**Figure 4:**
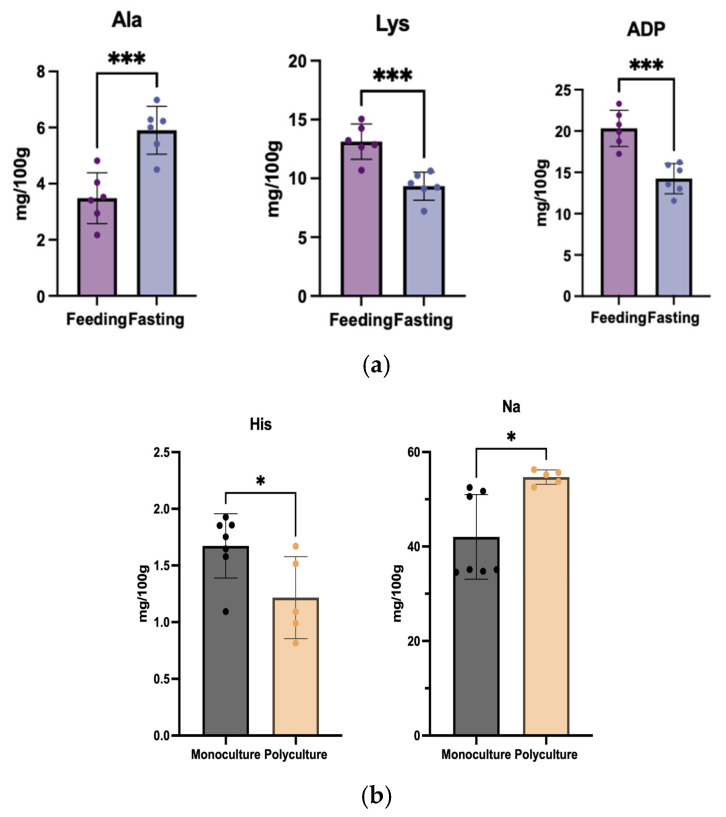
Analysis in *T. obscurus* for nutritional regime. (**a**) The contents of free amino acid, nucleotide, and inorganic ion between feeding and fasting regimens (*** *p* < 0.005, vs. raw); (**b**) the contents of free amino acid, nucleotide, and inorganic ion between monoculture and polyculture system (* *p* < 0.05, vs. raw).

The comparative metabolic profiling of *T*. *obscurus* under continuous feeding versus 5-day fasting regimes revealed distinct nutritional stress responses with nuanced implications for product quality and sensory characteristics. Fasted samples exhibited a 70% increase in Ala (5.74 vs. 3.38 mg/100 g, *p* < 0.01) (Figure 4a), indicative of gluconeogenesis activation during food deprivation. A pattern consistent with documented metabolic responses in other fish species, where transamination of branched-chain amino acids generates alanine to fuel hepatic gluconeogenesis [58,59]. Conversely, fed samples maintained 49% higher Lys (13.84 vs. 9.29 mg/100 g, *p* < 0.01), reflecting sustained mTORC1 signaling pathway activation [60], which enhances dietary amino acid utilization through ribosomal biogenesis regulation while suppressing lysosome-mediated essential amino acid catabolism, establishing a metabolic “sparing” mechanism. Additionally, nucleotide metabolism showed notable adaptive compartmentalization. ATP/ADP ratios were 52% higher in continuously fed groups (0.32 vs. 0.21, *p* < 0.01), reflecting more active energy metabolism. However, IMP levels remained stable between regimes (303.60 vs. 299.30 mg/100 g, *p* > 0.05), suggesting prioritization of umami-relevant nucleotide pools during short-term fasting. A multitude of studies have determined that adequate fasting prior to fish collection is advantageous in terms of enhancing its flavor and texture [61,62]. This discrepancy may arise from insufficient fasting duration to deplete odorous metabolites or trigger textural remodeling, coupled with compensatory mechanisms preserving core flavor compounds (IMP stability). Although alterations in amino acids and nucleotides were observed in *T*. *obscurus*, these variations did not manifest as perceptible differences in the sensory evaluation of the taste.

The comparative analysis between monoculture and polyculture in *T*. *obscurus* aquaculture revealed limited biochemical divergence (Figure 4b), with His and Na+ emerging as the sole significantly differentiated parameters. The content of His was found to be 28% higher in the monoculture group, which might be attributed to the absence of competitive factors in the environment, leading to the optimized nutrient partitioning toward species-specific metabolic pathways. In contrast, *T*. *obscurus* in the mixotrophic environment might have fostered conditions conducive to His synthesis due to its resilience to stress reactions. Conversely, Na^+^ content in the monoculture group was lower than that in the mixotrophic group, likely attributable to the enhanced osmoregulatory demands in multispecies environments to maintain osmotic pressure balance within the body. Mixed culture mode has also been identified as a prevalent practice in aquaculture, predominantly involving the cultivation of grass carp and bighead carp [63,64], which polyculture improves feed conversion efficiency and spatial optimization without compromising product quality.

This study revealed that the disparities between the mixed culture and monoculture groups were minimal, with no significant differences observed in sensory evaluation for *T. obscurus*. The findings advocate for expanded adoption of ecologically integrated aquaculture models that reconcile economic viability with biochemical quality preservation in commercial pufferfish production.

## 4. Conclusions

This comprehensive investigation provides the metabolic-sensory regulatory governing quality attributes in *T. obscurus*, revealing the biochemical composition determined by environmental and nutritional modulation through evolutionary-optimized adaptation mechanisms. As a result, the relationships across aquaculture factors, biochemical composition, and sensory characteristics were established through systematic analysis of seasonal variations, salinity effects, and nutritional regimes.

Seasonal metabolic reprogramming emerged as a critical determinant of quality optimization for *T. obscurus*. Spring samples exhibited 44.7% higher IMP and 92.8% elevated ATP compared to autumn, directly correlating with superior umami-kokumi attributes. These biochemical adaptations, extending beyond simple temperature responses, designate the post-overwintering period as a strategic harvest window, during which nucleotide and amino acid accumulation peaks, ensuring optimal flavor and nutritional quality for premium product development. Environmental salinity functioned as a master regulator of metabolic trade-offs, with a dichotomy of flavor-enhancing amino acids and sodium content. These results reflect an evolutionary balancing act between osmoregulatory efficiency and culinary quality optimization, enabling targeted environmental manipulation to align survival biochemistry with gastronomic excellence. Notably, short-term fasting induced distinct metabolic signatures without core sensory attributes, demonstrating remarkable nutritional plasticity that supports preharvest management strategies to reduce production costs while maintaining flavor integrity. The study further validated polyculture compatibility through minimal biochemical-sensory divergence from monoculture systems, establishing ecological aquaculture models as economically viable strategies that enhance resource efficiency without sacrificing quality standards. These results provide a scientific basis for evidence-based aquaculture management and quality control strategies.

To optimize flavor profiles and economic returns, we recommend maintaining brackish water salinity (3‰), as this regime selectively enriches umami-associated amino acids while ensuring osmoregulatory efficiency. Strategic harvesting should align with post-overwintering phases to capitalize on seasonal peaks in flavor-active compounds. Short-term fasting offers a cost-effective approach to reduce feed inputs without compromising sensory integrity. Furthermore, polyculture systems with herbivorous species demonstrate ecological and economic viability, achieving resource efficiency comparable to monoculture in both biochemical and sensory outcomes. In this study, long-term effects of seasonal adaptation on genetic or epigenetic regulation remain unexplored. Future research should integrate analyses with multi-omics approaches (e.g., transcriptomics, metabolomics) to unravel molecular mechanisms underlying observed phenotypic adaptations. Additionally, consumer acceptance trials are needed to validate sensory outcomes under real-market conditions.

## Figures and Tables

**Table 1:** Experimental design matrix for *T. obscurus* sampling.

Collection Season	Water Environment	Pre-Harvest Treatment	Group Code
Autumn	Freshwater (0‰)	Regular feeding	AUN
Autumn	Freshwater (0‰)	5-day fasting	AUY
Autumn	Saline (3‰)	Regular feeding(mixed)	ASN-M
Autumn	Saline (3‰)	5-day fasting(mixed)	ASY-M
Autumn	Saline (3‰)	Regular feeding	ASN
Autumn	Saline (3‰)	5-day fasting	ASY
Spring	Freshwater (0‰)	Regular feeding	SUN
Spring	Freshwater (0‰)	5-day fasting	SUY
Spring	Saline (3‰)	Regular feeding (mixed)	SSN-M
Spring	Saline (3‰)	5-day fasting (mixed)	SSY-M
Spring	Saline (3‰)	Regular feeding	SSN
Spring	Saline (3‰)	5-day fasting	SSY

**Table 2:** Overall range and distribution of biochemical parameters.

Parameter	Range	Mean	CV (%)
Moisture (%)	74.17–79.25	76.16	2.20
Ash (%)	3.38–4.39	3.84	8.45
Crude protein (mg/100 g)	17.60–22.46	19.50	8.17
Crude lipid (mg/100 g)	0.60–2.10	1.13	39.63
Asp (mg/100 g)	0.43–1.68	0.87	45.94
Glu (mg/100 g)	0.35–2.19	1.16	60.69
Ser (mg/100 g)	1.02–3.20	1.98	35.85
Gly (mg/100 g)	7.38–15.87	10.27	26.64
Thr (mg/100 g)	0.53–2.37	1.19	51.97
Ala (mg/100 g)	2.17–6.98	4.50	46.06
Pro (mg/100 g)	0.47–4.63	1.79	88.85
Lys (mg/100 g)	7.20–15.04	11.45	23.54
Trp (mg/100 g)	0.026–0.10	0.061	36.29
Val (mg/100 g)	0.38–1.67	0.93	45.06
Met (mg/100 g)	0.13–0.47	0.27	39.07
Phe (mg/100 g)	0.18–1.07	0.53	64.65
Ile (mg/100 g)	0.17–1.03	0.53	62.42
Leu (mg/100 g)	0.44–2.04	0.98	60.28
His (mg/100 g)	0.82–1.93	1.58	30.12
Arg (mg/100 g)	4.02–8.66	6.44	26.55
Tyr (mg/100 g)	0.046–0.14	0.099	31.60
CMP (mg/100 g)	2.01–4.40	3.18	26.64
GMP (mg/100 g)	12.56–20.47	15.33	16.17
IMP (mg/100 g)	220.48–398.78	288.53	19.70
ATP (mg/100 g)	2.73–9.04	4.20	49.72
ADP (mg/100 g)	11.56–23.31	16.80	26.94
AMP (mg/100 g)	1.02–5.32	2.89	52.17
HX (mg/100 g)	7.38–16.94	12.65	26.83
HXR1 (mg/100 g)	6.99–15.28	10.72	25.19
Na (mg/100 g)	34.55–56.29	47.14	15.83
K (mg/100 g)	170.28–307.80	236.29	17.65

CV = (σ/μ) × 100%, where σ = standard deviation, μ = mean value.

## Data Availability

The original contributions presented in the study are included in the article, further inquiries can be directed to the corresponding author.
